# A Noval Method for Surgical Removal of the Impacted Mandibular Third Molar: Sartawi Technique

**DOI:** 10.1155/2020/8876086

**Published:** 2020-09-25

**Authors:** Hassan Sartawi

**Affiliations:** Department of Dentistry, Al-Haramain Medical Complex, Riyadh, Ahad Rufaidah, Saudi Arabia

## Abstract

**Background:**

The goal of this article is to present and evaluate the clinical effectiveness of a new surgical approach using a triangular flap with slight modification and a 3-0 black braided silk surgical suture as flap retractor which is later used after the surgical procedure as a normal suture, aiming to decrease procedure time, soft tissue retraction, and tools for removal of impacted mandibular third molar.

**Methods:**

Patients requiring removal of fully impacted or semi-impacted lower third molars are treated with a new approach using minimal steps and tools, a simple triangular flap, slight mucoperiosteum elevation, as the flap sides are secured and reflected with a silk suture by an assistant holding both sides of the suture from behind the patient.

**Results:**

The surgical area at the procedure was efficiently exposed, and the separation of the crown from the roots was easily done using a surgical handpiece, separation and removal of the crown, removal of the roots with a straight elevator, without the need of flap retractor or overexposure of the surgical side with a conventional triangular flap or others. After the treatment, the two sides of suture are tied together with double overhand knots, and the surgical site was fully repositioned and closed without any complications. 5- and 7-day follow-up was done on the patients, and no complications were reported.

**Conclusions:**

This preliminary study presents a new surgical approach (Sartawi technique) which can be used during extraction of impacted and semi-impacted lower third molars, the results showed that the operation time was noticeably reduced, the size of exposed mucoperiosteum tissue was minimized compared to the conventional method, the use of the mucoperiosteum elevator was eliminated, and number of suture knots and suture used to close the surgical site reduced to a single stitch.

## 1. Introduction

Impaction is defined as the inability of a specific tooth to maintain its right position in the jaw due to malposition, lack of space, or other impediments [1]. Another definition is tooth that fails to erupt into the dental arch within the expected time [2]. In 2004, another definition was introduced by Farman: teeth that prevented from eruption due to a physical barrier with the path of eruption [3].

Despite major advances in the practice of dentistry, extraction of impacted third molars still carries risks of intra- and postsurgical complications. The compilation rate of 4.6-30.9% following the extraction of third molars is reported in the literature [4–8], which may occur intraoperatively or develop during the postoperative period.

An understanding of anatomical features of the surrounding structures and causes of extraction complications of the impacted tooth is important for the performance of proper extraction with minimal risk of complications. Extraction techniques using proper surgical protocols and correct technical approach permit efficient extraction procedures and decrease intraoperative complications which may include bleeding, damage to adjacent teeth, injury to surrounding tissues, displacement of teeth into adjacent spaces, fracture of the root, maxillary tuberosity, or the mandible. Postoperative complications may include swelling, pain, trismus, prolonged bleeding, dry socket, infection, and sensory alteration of the inferior alveolar nerve or lingual nerve.

The extractions of impacted mandibular third molars are one of the most common complaints that require surgical intervention [9, 10]. The aim of this preliminary study is to present a simplified version compared to the traditional techniques atraumatic as possible in minimal amount of time, which could lead to a significant impact on intra- and postsurgical complications.

## 2. Material and Methods

Study samples: a total of two patients suffering from mesioangular impacted left mandibular third molars were treated using this technique.

**Figure 1:**
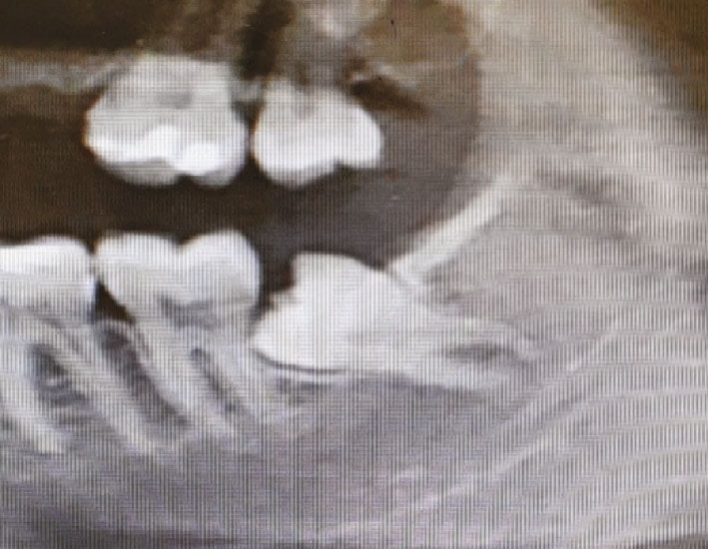
X-ray case 1.

**Figure 2:**
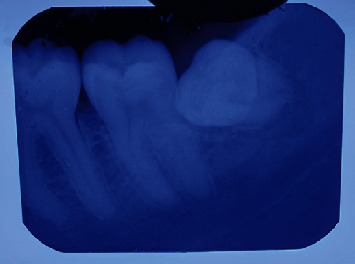
X-ray case 2.

The patients' cases were recorded and reported in this case study as follows. A 24 years old male patient (case 1) and 26-year-old male patient (case 2) presented to the clinic suffering from continuous pain in the mandibular left region, none of them reported any past medical history or systemic diseases, conventional X-ray examination (panoramic and periapical) showed mesioangular impaction of the left mandibular third molar with slight resorption in the distal root of the second molar (Figures 1 and 2), and both cases were indicated for surgical removal of the impacted third molar.

Signed consent forms were taken from the two patients, and the method was performed in accordance with the relevant guidelines and regulations.

Ethical approval was obtained from the Ethics Committee of Al-Haramain Medical Complex, Ahd Rofidah, Saudia Arabia.

### 2.1. Surgical Method

Step 1: anesthesia: inferior alveolar nerve block, buccal nerve block, lingual nerve block, and local infiltration for homeostasis in the surgical field with 2% lidocaine hydrochloride were administered (1 : 200000 epinephrine).

Step 2: gaining access to the impacted tooth: incision for a triangular flap extending to the middle buccal gingival sulcus of the mandibular second molar with surgical blade and slightly reflected from both incision sites enough to expose the crown using mucoperiosteum elevator.

**Figure 3:**
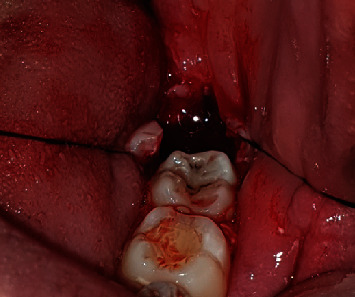
Intraoperative case 1.

**Figure 4:**
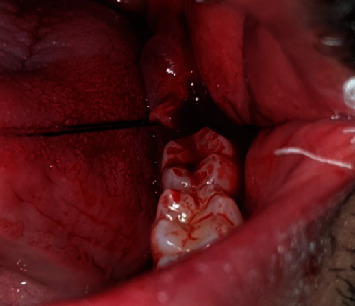
Intraoperative case 2.

Step 3: Sartawi's technique part 1: two 30 cm 3/0 silk surgical threads were used. One was inserted into the buccal side of the flap in the middle point of the flap line distally to the second molar and passed outside the mouth to the left side of the patient; meanwhile, the other was inserted into the lingual side of the flap in the middle point of the flap line and passed outside the mouth to the right side of the patient (Figures 3 and 4).

**Figure 5:**
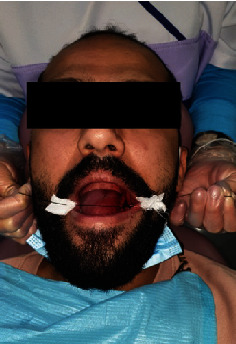
Intraoperative.

Step 4: Sartawi's technique part 2: two weaves of gauze were placed at both corners of the mouth to protect it from the pulling friction of the silk thread from behind the patient. The assistant pulled the threads from both sides to compensate for a flap retractor (Figure 5).

**Figure 6:**
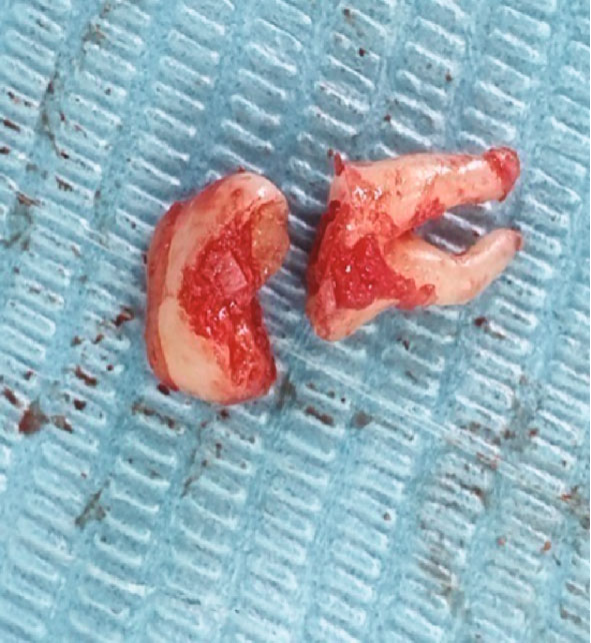
Extracted tooth case 1.

**Figure 7:**
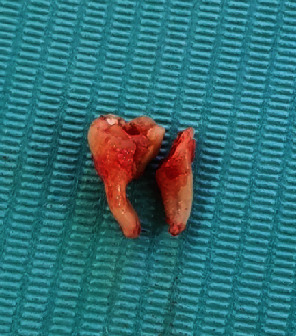
Extracted tooth case 2.

Step 5: bone Removal and tooth sectioning using surgical drill and a straight elevator used to luxate and remove the roots, the teeth were extracted (Figures 6 and 7), and the socket was irrigated with normal saline, and bony irregularities were corrected.

**Figure 8:**
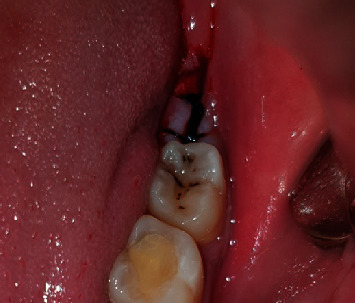
Postoperative case 1.

**Figure 9:**
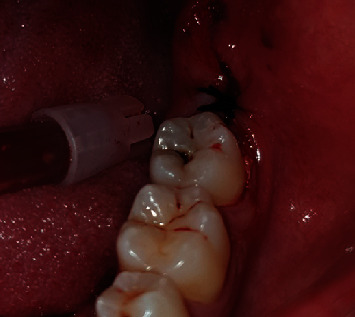
Postoperative case 2.

Step 6: Sartawi's technique part 3: both surgical threads mentioned in step 3 were tied in a double overhand knot causing complete closure of the surgical site, not requiring any further stitches (Figures 8 and 9).

Following the procedure, detailed postoperative instructions were given to the patients, and suitable antibiotics and analgesics were prescribed.

Slight postoperative bleeding was noticed immediately after the procedure was completed, which was managed with pressure packs.

### 2.2. Postoperative Follow-Up

**Figure 10:**
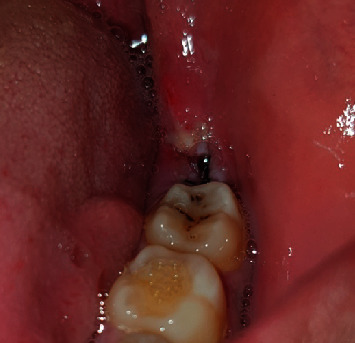
5 days follow-up case 1.

**Figure 11:**
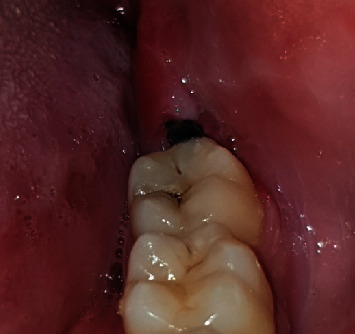
5 days follow-up case 2.

Both patients presented to the clinic 5 days after the surgical procedure for the follow-up process. and none of them reported any complications; except in case one, patient reported white discoloration at the surgical site and was found to be a slight food particle stagnation (Figures 10 and 11).

at 7 days which both patients presented for removal of the stitches, and complete tissue healing was noticed.

## 3. Discussion

There are several intraoperative and postoperative complications that might occur during and after the extraction of the impacted mandibular third molar which can be reduced by understanding the possible causes and how to prevent each of these complications; the Sartawi technique focused on four major causes and ways to prevent it, and those four major causes are instruments, flap design, suture stitches, and time.

Less the number of oral surgical instruments, such as flap retractor, used in oral cavity operations would decrease the possibility of tissue trauma and buccal and lingual nerve damage [11, 12] as well as decreasing the possibility of infection caused by the instruments used, and the Sartawi technique limits the abovementioned risks as the flap retractor is totally eliminated from the surgical procedure.

There are variable styles of flap design used in the removal of the impacted mandibular third molar, mainly envelop and triangular flaps, and their modification has been developed to minimize those complications; the triangular flap design is associated with patients consuming the least pain killer [13]; therefore, in the Sartawi technique, the flap used was a triangular flap with a slight modification that leads to minimize mucoperiosteum elevation.

Number of stitches has a significant effect on the postsurgical complications, where more stitches can lead to accumulation of food, causing infections and halitosis. There are no specific data available on the correlation between the number of stitches/knots and their effect on wound healing; however, barbed suture (knotless) is considered a safe and efficient alternative to conventional stitches for the suturing of free flaps to the local tissue [14], thereby it can be quoted that using lesser knots leads to better healing and consecutively lesser complications; in the Sartawi technique after the suture is used as a flap retractor, the two sides are brought together in a double overhand knot and cause complete closure of the surgical flap with a single double knot stitch.

The time of the operation and postsurgical complications have a direct correlation, where increasing the operating time is associated with more postoperative morbidity [15], and the duration of surgery affected the acute postoperative symptoms and signs after the lower third molar extraction [16], and in the Sartawi technique, the time of the surgical operation is reduced dramatically which leads to less intra- and postsurgical complications.

Nevertheless, there are some limitations and drawbacks for the Sartawi technique, mainly the need for a second assistant as the first one's hands are full holding the threads retracting the flap. The operating field is smaller compared to the conventional method and causes less control for the surgeons performing it and can only mastered if the surgeon is well experienced with the conventional methods. The study samples were limited to 2 patients with mesioangular impaction and was not performed on patients with other types of anatomical impaction positions.

## 4. Conclusion

According to this preliminary study, the use of this technique was tolerated by the two patients, and they did not have any intra- or postoperative complications; the steps are easily done by an experienced oral surgeon familiar with the conventional surgical protocols of impacted mandibular third molar surgery; however. larger studies are needed to evaluate significance and the quality of this technique as this technique is worthy of clinical promotions.
